# Surface Plasmon Resonances in Sierpinski-Like Photonic Crystal Fibers: Polarization Filters and Sensing Applications

**DOI:** 10.3390/molecules25204654

**Published:** 2020-10-13

**Authors:** William O. F. Carvalho, J. R. Mejía-Salazar

**Affiliations:** National Institute of Telecommunications (Inatel), 37540-000 Santa Rita do Sapucaí, MG, Brazil; william.carvalho@dtel.inatel.br

**Keywords:** fractal geometry, refractive index sensor, surface plasmon resonance (SPR), photonic crystal fiber (PCF), polarization filter

## Abstract

We investigate the plasmonic behavior of a fractal photonic crystal fiber, with Sierpinski-like circular cross-section, and its potential applications for refractive index sensing and multiband polarization filters. Numerical results were obtained using the finite element method through the commercial software COMSOL Multiphysics^®^. A set of 34 surface plasmon resonances was identified in the wavelength range from λ=630 nm to λ=1700 nm. Subsets of close resonances were noted as a consequence of similar symmetries of the surface plasmon resonance (SPR) modes. Polarization filtering capabilities are numerically shown in the telecommunication windows from the O-band to the L-band. In the case of refractive index sensing, we used the wavelength interrogation method in the wavelength range from λ=670 nm to λ=790 nm, where the system exhibited a sensitivity of S(λ)=1951.43 nm/RIU (refractive index unit). Due to the broadband capabilities of our concept, we expect that it will be useful to develop future ultra-wide band optical communication infrastructures, which are urgent to meet the ever-increasing demand for bandwidth-hungry devices.

## 1. Introduction

Surface plasmon resonances (SPRs) are enhanced electromagnetic fields bound to metal/dielectric interfaces through resonantly coupled optical and electronic excitations [1]. Due to their myriad applications in sub-diffraction nanophotonic devices for imaging, communications, energy harvesting, and sensing/biosensing applications, SPRs are gaining considerable attention during the last years [2,3,4,5,6,7,8,9,10,11]. In particular, SPRs allow for real-time and label-free monitoring of molecular-binding events (near the interface) through detection of small changes in the local refractive index [12,13,14,15]. Furthermore, high-performance polarization beam splitters and filters, of great interest for modern optoelectronics and communication systems, can also be designed and developed based on the SPR excitation principle [16,17]. Among the variety of strategies for SPR excitation, photonic crystal fibers (PCFs) are deserving special attention because of their unique features like high flexibility, birefringence, wide tuning range, good temperature stability, and a proper handling of the evanescent fields [17,18,19,20]. The cross-section of these SPR-PCFs are usually made by periodic arranges of air holes, some of which are filled or coated with metallic components, surrounding one or more cores (where light is confined). The SPR excitation is thus reached in PCFs through the phase-matching between the core-mode and the surface plasmon mode, i.e., when both modes have the same effective refractive indices [21,22,23,24,25,26,27,28,29,30,31,32,33]. In spite of the extensive research efforts on plasmonic PCFs, the narrow frequency range for SPR excitation constitutes a major hurdle for practical applications in, for example, sensors and multiband polarization beam splitters and filters [16,17,30,32].

The use of fractal geometries, on the other hand, has been successfully exploited during the last decades for the realization of multiband (or broadband) compact and high-performance antennas [34,35,36]. More recently, these self-similar geometries have also enabled multispectral compatibility and multiple applications when used for patterning one- and two-dimensional plasmonic superlattices [37,38,39,40,41]. In contrast, self-similar plasmonic properties in SPR-PCFs remain unexplored. Herein, we demonstrate the excitation of multiple SPRs in PCFs with fractal cross-section designs. To this end, we utilized a Sierpinski-like [42] geometry for the corresponding circular cross-section of the fiber, where one of the subsets in the fractal geometry was considered with metallic inclusions. Importantly, using this fractal design we found a set of 34 plasmonic modes in the frequency range from 630 nm to 1700 nm, which enable applications from the visible to the infrared regime. These multiple resonances are explained by self-similar effects, which as recently demonstrated for triangular Sierpinski fractals are due to self-similar hierarchy of metallic scatterers in the structure [38,41]. Calculations in this work were made using the finite element method (FEM), through the commercial software COMSOL Multiphysics^®^. Potential applications for high-performance refractive index sensing and for multiband polarization filters are also shown here.

## 2. PCF Design and Modeling

**Figure 1:**
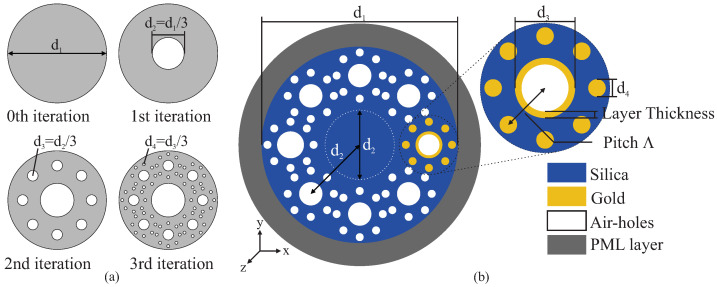
(**a**) Pictorial representation of the generation of a Sierpinski-like circular cross-section. (**b**) The Sierpinski-like cross-section used in the surface plasmon resonance (SPR)-photonic crystal fiber (PCF). The inset is used to show the geometry of the plasmonic fractal subset and to identify different materials considered in the simulations. The center circle, indicated by a dashed circle, corresponds to the dielectric fiber core.

In Figure 1a we schematized the design of the fractal cross-section of the optical fiber. A solid circle of diameter $d_{1}$ is used as the starting point, i.e., as the 0-th iteration step. The solid circle is then divided into 9 identical circles with fractional diameter $d_{2}=\frac{d_{1}}{3}$. More specifically, these sub-circles are distributed as a central circle surrounded by eight identical circles. The central circle is removed in the first iteration step and, as depicted in Figure 1a, the same procedure is recursively applied to the remaining eight solid sub-circles and so on until the third iteration step. We used this geometry to build our fractal PCF but without removing the center circle (fiber core), where light is guided along the fiber, indicated by a dashed circle in Figure 1b. Plasmonic effects are introduced in the system considering that one of these fractal subsets has metallic components. In particular, we considered a fractal subset consisting of a gold-coated hole surrounded by gold nanowires, as represented in the inset of Figure 1b.

Numerical results in this work were obtained with FEM simulations using the commercial software COMSOL Multiphysics^®^. In order to avoid numerical reflections at the edge of the structure, we considered a circular perfectly matched layer (PML) in addition to scattering boundary conditions around the optical fiber. An optimized mesh-size was also used for accurateness in the numerical results. Calculations were made using $d_{1}=9.9$ μm, $d_{2}=3.3$ μm, $d_{3}=1.1$ μm, and $d_{4}=0.367$ μm. Polarization is defined according to the orientation of the electric field of light respect to the coordinate system in Figure 1. *x*-polarized or *y*-polarized are used to indicate that the electric field is considered oscillating along the *x*- or *y*-axis, respectively. The holes in the fiber structure are considered to be filled with air, i.e., with a refractive index $n_{\mathrm{holes}}=1$. The experimental results for the permittivity of gold in Ref. [43] were used for metallic components in the system. The PCF is considered made of fused silica, for which we used the refractive index according to the well-known Sellmeier equation [44]

(1) $$n\left(\lambda \right)=\sqrt{1+\sum_{m=1}^{3}\frac{B_{m}\lambda^{2}}{\lambda^{2}-C_{m}}},$$

where $B_{1}=0.696163$, $B_{2}=0.4079426$, $B_{3}=0.8974794$, $C_{1}=0.00467914826$, $C_{2}=0.0135120631$, and $C_{3}=97.9340025$ are the Sellmeier coefficients for fused silica [44] and λ is the wavelength of light in μm. We used 50 nm for the layer thickness in Figure 1b.

An important parameter for the analysis is the confinement loss (α), defined as

(2) αx,y(λ)=8.686k0×Im[neff(λ)]×104dB/cm,

where $k_{0}=2\pi /\lambda$ is the wave vector in free space, Im[$n_{eff}$] is the imaginary part of the core-guided mode effective refractive index. Under the phase-matching condition, Im[$n_{eff}$] is considerable enhanced due to the resonant coupling between the core-guided mode and the SPR mode [22]. Therefore, changes in α can be used to monitor the SPR excitation in the structure. The subindexes *x* and *y* in Equation (2) are used to indicate the corresponding polarization of light. Since we are using a fiber cross-section that lacks of mirror symmetry around the *y*-axis, see metallic wires on the right-hand side of Figure 1b, we expect $\alpha_{x}\neq \alpha_{y}$, which is commonly known as a birefringence effect. Indeed, this last property can be exploited as an efficient mechanism for SPR-based polarization filtering applications [24,29]. Previous reports on this important application have been mostly focused on gold-coated air holes [17,21,22,30], gold nanowires [31] and D-shaped [32] structures.

## 3. Results and Discussion

**Figure 2:**
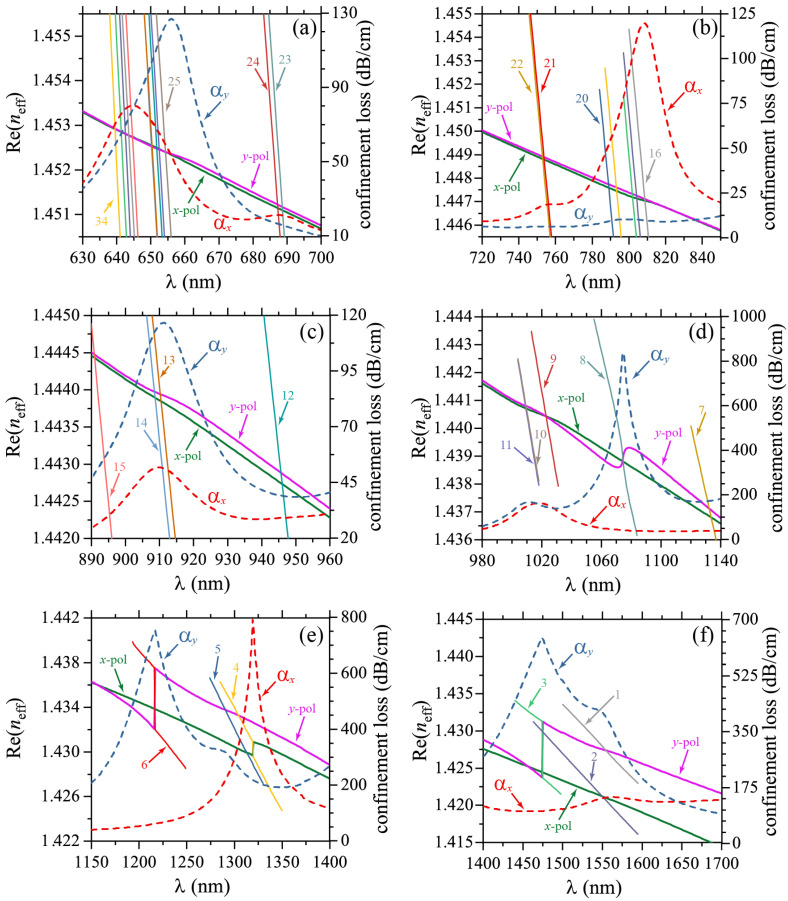
The Re(neff), for the SPR and fiber modes, and confinement loss ($\alpha_{x,y}$) are plotted in the wavelength range from λ=630 nm to λ=1700 nm. A large set of SPR modes was identified which, for visualization purposes, were numbered from 34 to 1 and presented in (**a**) 34 to 23, (**b**) 22 to 16, (**c**) 15 to 12, (**d**) 11 to 7, (**e**) 6 to 4 and (**f**) 3 to 1.

**Figure 3:**
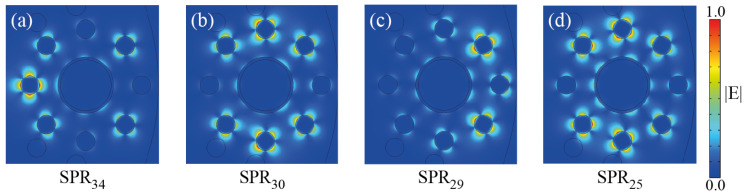
Plasmonic field profiles of SPR modes from Figure 2. Results are for (**a**) SPR${}_{34}$, (**b**) SPR${}_{30}$, (**c**) SPR${}_{29}$, and (**d**) SPR${}_{25}$, where the subindex indicate the corresponding mode number in Figure 2.

The circular symmetry of each metallic nanowire and nanoshell allows for a large set of plasmonic resonaces of different orders [45]. Moreover, the interaction between nearby metallic scatterers increases the number of allowed resonances through plasmonic hybridization [46,47], i.e., the overlap of plasmonic near-fields between adjacent metallic scatterers, which resembles the electronic bands from well-localized atomic orbitals in solid state physics [48,49]. The fractional sizes of different nearby scatterers, i.e., the fractal-like geometry, also introduce a broadening of the frequency range for SPR excitation [38,41]. In Figure 2, we plot the Re(neff) and $\alpha_{x,y}$-values (confinement loss) for λ ranging from 630 nm to 1700 nm. For visualization purposes, this wavelength range was divided in six different regions presented in Figure 2a–f. Results of Re(neff) for the SPR and core-guided modes are shown for *x*-pol and *y*-pol. From the phase-matching condition we identified a total of 34 SPR modes in the structure, as it can be seen from Figure 2, which were labeled with numbers from 34 to 1. For small λ values we can note subsets of close SPR modes, due to similar symmetries of the SPR modes [50], which must overlap producing broader peaks. In Figure 3, we show the near-field profiles of SPR${}_{34}$, SPR${}_{30}$, SPR${}_{29}$, and SPR${}_{25}$, from where we clearly note that the SPR modes preserve the mirror-symmetry of the structure around the *x*-axis, i.e., for rotations of 180${}^{\circ }$, as expected. We also note two pair of degenerate SPR modes, in particular the modes 21 and 22, and 10 and 11, which are due to equivalent high-order symmetries of the circular geometry [46,47]. We should remark the polarization selective excitation of SPR modes. The weakly confined modes labeled as 23 and 24 in Figure 2 are only excited for the *x*-polarization, where a small peak for $\alpha_{x}$ is observed around the phase-matching condition. Moreover, in the frequency range from 720 nm to 850 nm, plasmon resonances are exclusively excited for *x*-polarization. This polarization selectivity becomes more evident for higher λ values, as noticed from Figure 2e,f.

**Figure 4:**
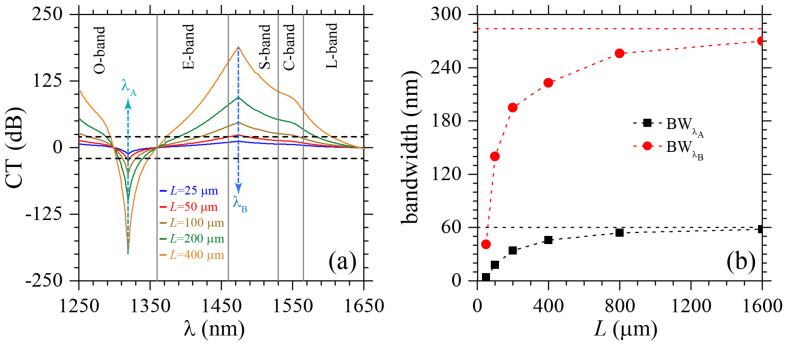
(**a**) Cross-talk parameter as function of the wavelength. Results are presented in the wavelength range from 1250 nm to 1625 nm, i.e., from the telecommunications O-band to the L-band, for different fiber lengths *L*. The horizontal dashed lines are used to indicate the threshold values of the cross-talk (CT) parameter, i.e., ±20 dB CT, for polarization filtering applications. The vertical dashed arrows show the resonant wavelengths (phase-matching condition), where maximum and minimum peaks of CT are observed for all *L* values. Solid vertical lines help visually identify different telecommunication bands. (**b**) The bandwidths for polarization filtering, at wavelengths $\lambda_{A}$ (BW_A_) and $\lambda_{B}$ (BW_B_), is presented as function of the fiber length. BW_A_ and BW_B_ asymptotically approach 60 nm and 284 nm, respectively, as indicated by horizontal dashed lines.

Let us now discuss the potential use of the proposed SPR-PCF in polarization filtering applications. To this end, we used the polarization cross-talk (CT) parameter, i.e., the transmission performance according to $\alpha_{x}$ and $\alpha_{y}$, as [33]

(3) $$\mathrm{CT}\left(\lambda \right)=20\log\left(\exp[\left(\alpha_{y}-\alpha_{x}\right)L]\right)\;\mathrm{dB},$$

where *L* is the fiber length. Since *x* and *y* polarizations can be easily separated for $\left|\mathrm{CT}\right|>20$ [33], we used ±20 dB as the reference values, indicated by horizontal dashed lines in Figure 4a. As the CT values strongly depend on *L*, see Equation (3), we carried out calculations for *L* = 25 μm, 50 μm, 100 μm, 200 μm and 400 μm. We should emphasize here that negative CT-values correspond to high *y*-pol signal over *x*-pol, whereas positive CT denote high *x*-pol signal over *y*-pol. From Figure 4a, we can see that polarization filtering only works for L>50 μm, i.e., when $\left|\mathrm{CT}\right|>20$. Two resonant wavelengths at $\lambda_{A}=1319$ nm and $\lambda_{B}=1474$ nm, corresponding to SPRs, are indicated by vertical dashed arrows in this figure. In terms of telecommunication windows, *x*-pol is filtered at the O-band (1260 nm to 1360 nm), whereas the *y*-pol is filtered at the E/S/C/L-bands (1360 nm to 1625 nm). The filtering bandwidths, associated to resonant wavelengths $\lambda_{A}$ and $\lambda_{B}$, are presented as BW${}_{A}$ and BW${}_{B}$, respectively, in Figure 4b for PCF lengths up to L=1600 μm. From this latter figure we clearly note a monotonically increasing of BW${}_{A}$ and BW${}_{B}$, which asymptotically approaches BW${}_{A}=60$ nm and BW${}_{B}=284$ nm.

**Figure 5:**
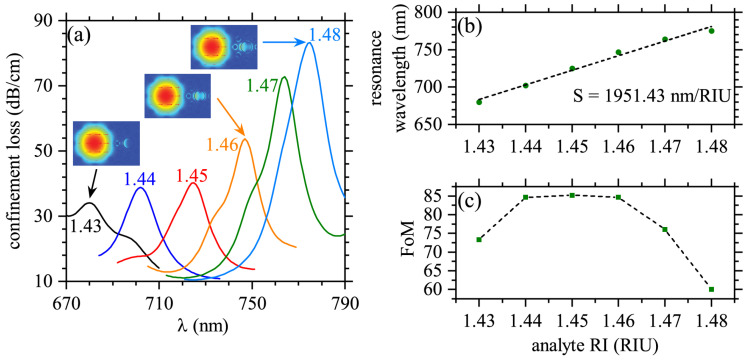
(**a**) Confinement loss for *x*-polarized light ($\alpha_{x}$) is presented as function of the wavelength for different refractive indexes of the analyte medium. The electric field profiles are shown in the insets for some peaks, as indicated by arrows. The corresponding refractive index of the analyte is shown in the corresponding $\alpha_{x}$ peak. (**b**) Wavelength of the peak in $\alpha_{x}$ as function of the refractive index of the analyte medium. Dashed line corresponds to the linear fitting of the results, with a slope (sensitivity) of 1951.43 nm/refractive index unit (RIU). (c) A FoM defined as the ratio between the sensitivity-peak and the corresponding full width at half maximum (FWHM), $\mathrm{FoM}=\frac{S(\lambda_{\mathrm{peak}})}{FWHM}$.

The second potential application we want to illustrate corresponds to a SPR-PCF-based refractive index sensor. It is well known that plasmonic sensing detects small changes of the refractive index near the metal/dielectric interface [4]. Therefore, we considered that the analyte can be flowing in a liquid or gas medium through the gold-coated hole in Figure 1b. Detection is performed by measuring the small shiftings in the resonant wavelengths, peaks in $\alpha_{x}$, due to small variations in the refractive index of the analyte medium. As shown in Figure 5a, we use the working wavelengths in the range from λ=670 nm to λ=790 nm, whilst the corresponding refractive index for the analyte medium was taken in the range from $n_{a}=1.43$ to $n_{a}=1.48$. The sensing performance, in the wavelength interrogation method, is calculated by [25]

(4) $$S\left(\lambda \right)=\frac{\Delta \lambda_{\mathrm{peak}}}{\Delta n_{a}},$$

where $\Delta \lambda_{\mathrm{peak}}$ is the wavelength shift of the peak in $\alpha_{x}$ associated to a change $\Delta n_{a}$ in the refractive index of the analyte medium. An important parameter to determine the reliability of sensor measurements is the sensing resolution, defined as

(5) $$R=\frac{\Delta n_{a}\times \Delta \lambda_{\min}}{\Delta \lambda_{\max}},$$

where $\Delta n_{a}$ is the analyte refractive index change, Δλ_min_ is the lower spectral resolution and Δλ_max_ is the higher resonant wavelength shift analyzed. S(λ) and *R* have dimensions of nm/RIU (refractive index unit) and RIU, respectively.

Results in Figure 5b are for the peaks in $\alpha_{x}$ as function of the refractive index of the analyte medium. The corresponding field-profiles illustrating the core-mode and SPR-mode for some $\lambda_{\mathrm{peak}}$ and $n_{a}$ are presented as insets in Figure 5a. Using a linear fitting, see the dashed line in Figure 5b, we obtained a sensitivity value of S(λ)=1951.43 nm/RIU. In addition to large S(λ) values, our system exhibited higher performance than other recent proposals [51,52,53], as it can be noted from the figure of merit, $\mathrm{FoM}=\frac{S(\lambda_{\mathrm{peak}})}{FWHM}$, shown in Figure 5c. The partial results for the performance, resonant wavelengths, $\lambda_{\mathrm{peak}}$, and S(λ) and *R* values are summarized in Table 1, in comparison with their average values at the last row. Because several experimental approaches to develop these types of SPR-PCFs are available, though the experimental realization may be challenging, we expect that the ideas presented here will stimulate exploitation of multiple SPRs for sensing and multiband polarization filtering applications.

## 4. Conclusions

In summary, the potential for sensing and multiband polarization filtering applications of plasmonic photonic crystal fibers, with Sierpinski-like cross-section, have been numerically demonstrated. The fractal fiber exhibited broadband capabilities, with a large set (34 modes) of plasmonic resonances from the visible (λ=630 nm) to the infrared (λ=1700 nm). The sensing performance was also evaluated, using the wavelength interrogation, with a linear sensitivity of S(λ)=1951.43 nm/RIU. Based on our results, we expect that further improvements can be made using other geometries like higher Sierpinski-steps, other fractal sequences, or different geometrical sizes of the holes and wires in the structure in order to tune the corresponding frequency ranges.

## Figures and Tables

**Table 1:** A comparison of different parameters for the sensing performance from results in Figure 5. Results are compared for successive refractive index steps and with the corresponding average.

Analyte RI	Res. Peak Wave. [nm]	Peak Loss [dB/cm]	Res. Peak Shifting [nm]	Wave. Sensitivity [nm/RIU]	Wave. Resolution [RIU]
1.43	680	34.12	–	–	–
1.44	702	38.08	22	2200	4.5454 × 10^−5^
1.45	725	40.27	23	2300	4.3478 × 10^−5^
1.46	747	53.68	22	2200	4.5454 × 10^−5^
1.47	764	72.82	17	1700	5.8823 × 10^−5^
1.48	775	83.20	11	1100	9.0909 × 10^−5^
Average	–	–	95	1900	5.2631 × 10^−5^
